# A simple, fast and reliable scan-based technique as a novel approach to quantify intracellular bacteria

**DOI:** 10.1186/s12866-019-1625-1

**Published:** 2019-11-12

**Authors:** Meysam Sarshar, Daniela Scribano, Giulia Tranquilli, Marisa Di Pietro, Simone Filardo, Carlo Zagaglia, Rosa Sessa, Anna Teresa Palamara, Cecilia Ambrosi

**Affiliations:** 1 Department of Public Health and Infectious Diseases, Sapienza University of Rome, Laboratory affiliated to Institute Pasteur Italia-Fondazione Cenci Bolognetti, 00185 Rome, Italy; 2 Microbiology Research Center (MRC), Pasteur Institute of Iran, Tehran, 1316943551 Iran; 3 Department of Public Health and Infectious Diseases, Sapienza University of Rome, 00185 Rome, Italy; 4 Dani Di Giò Foundation-Onlus, Rome, Italy; 5 IRCCS San Raffaele Pisana, Department of Human Sciences and Promotion of the Quality of Life, San Raffaele Roma Open University, 00166 Rome, Italy

**Keywords:** Intracellular bacterial quantification, In-cell Western, High-throughput, CFU, IFU, Immunofluorescence

## Abstract

**Background:**

Quantification of intracellular bacteria is fundamental in many areas of cellular and clinical microbiology to study acute and chronic infections. Therefore, rapid, accurate and low-cost methods represent valuable tools in determining bacterial ability to persist and proliferate within eukaryotic cells.

**Results:**

Herein, we present the first application of the immunofluorescence In-Cell Western (ICW) assay aimed at quantifying intracellular bacteria in in vitro infection models. The performance of this new approach was evaluated in cell culture infection models using three microorganisms with different lifestyles. Two facultative intracellular bacteria, the fast-growing *Shigella flexneri* and a persistent strain of *Escherichia coli*, as well as the obligate intracellular bacterium *Chlamydia trachomatis* were chosen as bacterial models. The ICW assay was performed in parallel with conventional quantification methods, i.e. colony forming units (CFUs) and inclusion forming units (IFUs). The fluorescence signal intensity values from the ICW assay were highly correlated to CFU/IFUs counting and showed coefficients of determination (R^2^), ranging from 0,92 to 0,99.

**Conclusions:**

The ICW assay offers several advantages including sensitivity, reproducibility, high speed, operator-independent data acquisition and overtime stability of fluorescence signals. All these features, together with the simplicity in performance, make this assay particularly suitable for high-throughput screening and diagnostic approaches.

## Background

Some bacterial pathogens have evolved a complex interplay with the host mediated by bacteria-derived factors. These factors enable bacteria to colonize and invade host cells as well as evade host immune systems, thereby representing the hallmark of pathogenesis [1–6]. According to their evolutionary adaptation, intracellular bacteria can be facultative or obligate [7–9]. Quantification of intracellular bacteria is the first approach in understanding pathogenicity factors involved in bacterial internalization and survival within host cells [6, 10]. Intracellular bacteria are largely studied through the cell culture infection models, in which eukaryotic cell monolayers are infected with the selected bacterial species. For facultative intracellular bacteria, the medium is supplemented with an antibiotic impermeable to eukaryotic cell membrane after the contact period, to kill extracellular bacteria, thereby allowing the exclusive selection of internalized bacteria [2, 8, 10]. The main method used to quantify facultative intracellular bacteria is to lyze infected cell monolayers using a low concentrated detergent. The cell lysates were then serially diluted and spot-plated for colony forming units (CFUs) count [5, 11]. This conventional technique has serious drawbacks, such as the incomplete lysis of eukaryotic cells, the type of detergent used for cell lysis that could interfere with bacterial viability, the choice of appropriate dilution for counting, the unsuitability for high-throughput analyses, the variable incubation times and the coalescence of colonies, leading to unreliable and ambiguous results [5, 12, 13]. Moreover, preparation of media, dilution tubes, as well as counting of colonies are time-consuming and labor-intensive [5, 12, 13]. Additionally, the CFU counting technique cannot be applied for obligate intracellular pathogens. Consequently, several alternative quantification methods, such as colorimetric and fluorescence-based methods have been developed [5, 14–21]. These techniques are mainly used for the quantification of obligate intracellular pathogens, such as *Chlamydia trachomatis*. This is usually achieved by counting the number of membrane-bound vacuoles (inclusions), expressed as chlamydial inclusion forming units (IFUs), using direct immunofluorescence [18, 22, 23]. Although, it provides high sensitivity and specificity, this method is time-consuming, tedious and requires extensive operator’s technical skills. Furthermore, it is particularly challenging for high-throughput assays.

The In-Cell Western (ICW) assay is based on the quantification of immunofluorescence signals from proteins in fixed cultured cells using target-specific primary antibodies. Gene expression levels, phosphorylation of proteins, caspases activity, cell proliferation assay and viral titration represent the common application of this assay [24–28]. Herein, we present the first application of this assay aimed at quantifying intracellular bacteria. The accuracy and efficiency of this extended application of the ICW-based assay was tested on two facultative intracellular pathogens, the fast-growing *Shigella flexneri* strain M90 T and the persister *Escherichia coli* strain LF82, as well as the obligate intracellular pathogen *C. trachomatis* L2 strain 434/Bu. The basic principle of this technique is to sequentially immunostain intracellular bacteria in infected cell monolayers seeded into multiwell plates with species-specific primary antibodies and appropriate infrared (IR) conjugated secondary antibodies, followed by quantification of the fluorescence signal intensity by the Odyssey CLx imager. Validation of this technique was achieved by comparison with standard quantification approaches. The proposed technique offers several advantages over current methods. In particular, it is a sensitive, scan-based, high-throughput method, allowing the analysis of up to 576 wells in a working day. No fluorescent tagged bacteria are required for the analysis, thereby widening the use of this technique to all bacteria for which there is availability of a primary antibody. Samples are minimally manipulated leading to more reproducible data. Additionally, the low autofluorescence background and advanced signal stability of IR dyes offer convenient, stable and accurate data. Lastly, quantification is not operator-dependent allowing the development of standardized protocols, opening its use for diagnostic purposes.

## Results and discussion

### Selection of primary and secondary antibody dilution factors for optimal ICW performance

**Fig. 1:**
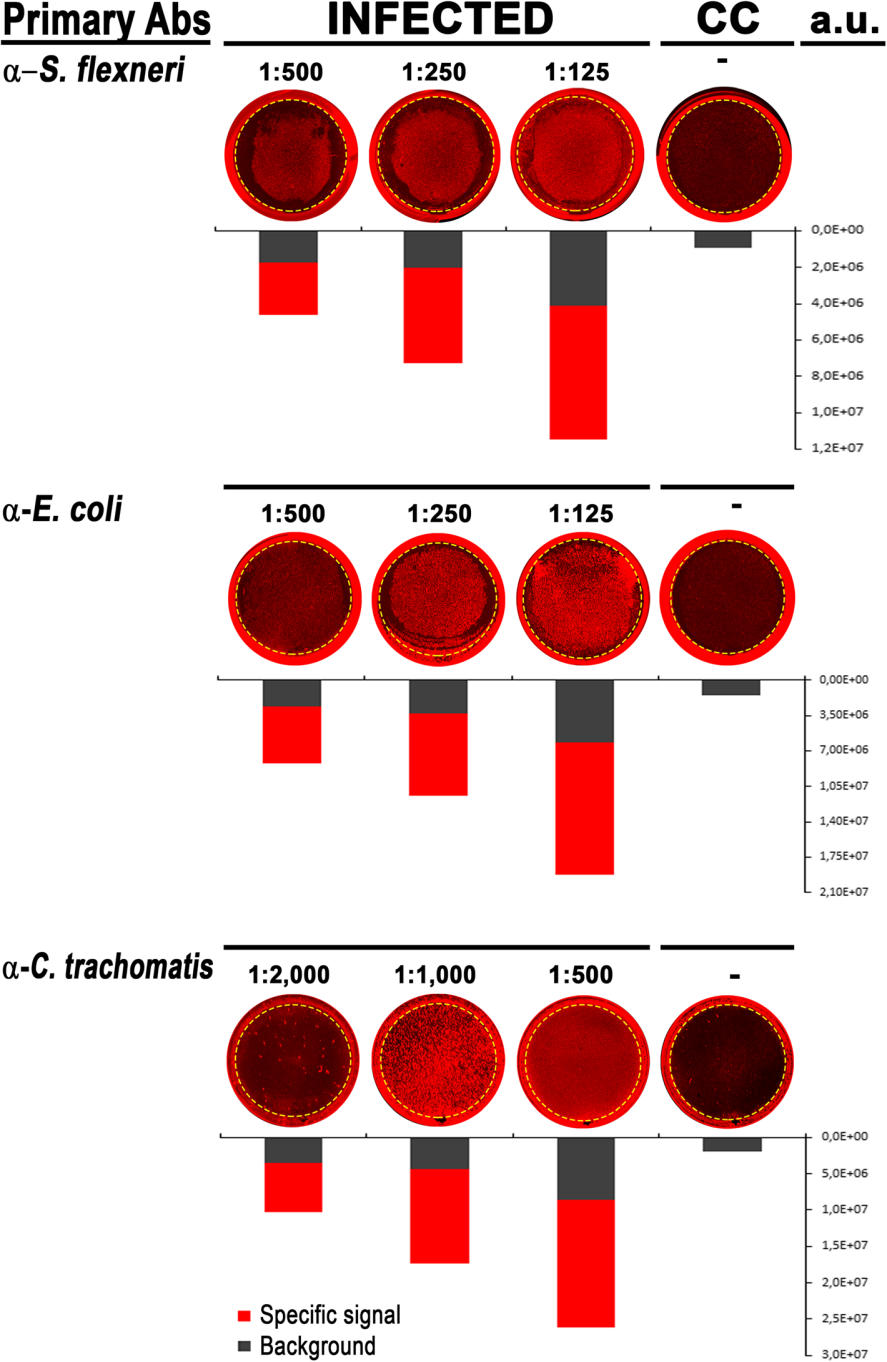
Setting the appropriate primary antibody concentrations for ICW assay. Cell monolayers were individually infected with *S. flexneri* (strains M90 T), *E. coli* (strain LF82) and *C. trachomatis* (strain 434/Bu), for 1 h, 24 and 36 h, respectively. Non-infected control cells (CC) were used as control. Primary antibodies were diluted as indicated, while the secondary antibody was used at 1:800. The bars below representative images indicate the a.u. mean values of specific antibody signals from infected monolayers (red) and from the background of non-infected control cells (black) from three independent experiments performed in quadruplicate. The a.u. values of non-infected cells stained only with the secondary antibody are shown. Dashed yellow rings mark the analyzed areas. Standard deviation (SD), not shown, was below 5% for the entire dataset

The ICW assay consists in an immunofluorescence-based technique in which the antibodies represent the only reagents that need to be optimized. For this purpose, antibody titration tests were performed by varying the dilution factors while keeping incubation time and temperature conditions fixed. Initially, we assessed the background values of secondary antibodies on semi-confluent non-infected HeLa cell monolayers. Following fixation and permeabilization, three different 2-fold serial dilutions of the commercially available anti-rabbit, anti-goat and anti-mouse secondary antibodies (1:200, 1:400 and 1:800), conjugated with two different IR fluorescent dyes (IRDye 680, red, and IRDye 800, green), were tested. Plates were analyzed using the Odyssey CLx Imaging System and arbitrary unit (a.u.) values were measured from 24-well standard scan grid enclosing the flat surfaces of the plates, as depicted by the yellow rings in Fig. 1. The highest dilution factor (1:800) showed minimal background signal and, therefore, was used throughout this study.

Two facultative intracellular pathogens, the human diarrheal pathogen *S. flexneri* serotype 5 strain M90 T and the adherent-invasive *E. coli* (AIEC) strain LF82, were chosen to assess the performance of the ICW assay. Thus, to optimize the appropriate concentration of their primary antibodies, infection assays using either the M90 T or the LF82 strains were performed (see the Methods section for details). After the proper time of gentamicin treatment, infected cell monolayers were fixed, permeabilized and immunostained with three different 2-fold serial dilutions of either rabbit anti-*S. flexneri* or goat anti-*E. coli* antibodies. Following hybridization with the appropriate secondary antibody IRDye 680-conjugated (referred to as IRDye 680), plates were scanned and analyzed. Both antibodies appeared to be specific in recognizing their target antigens in infected cells, providing a.u. values dependent on the dilution factors used (Fig. 1). To evaluate background signals, non-infected cells were hybridized with the three different dilutions of strain-specific primary antibodies followed by the IRDye 680 secondary antibody (1:800); thus, the a.u. values were subtracted from those measured for infected cells. The a.u. values recorded for both antibodies in non-infected cells were very low, indicating that background signals arising from non-specific interactions were negligible (Fig. 1). Among those tested, the dilution factor of 1:250 provided the best staining results, being the one that gave the highest a.u. values with minimal background and, therefore, was used throughout this study (Fig. 1).

The third microorganism model included in this study to assess the performance of the ICW assay was the intracellular obligate bacterium *C. trachomatis* responsible for genital tract diseases. To allow the comparison between IFU and a.u. quantification methods without any technical bias related to the efficiency of the primary antibody, the same mouse monoclonal antibody anti-*Chlamydia* lipopolysaccharide (LPS) routinely used for IFU counting was chosen, but in the unconjugated form. To optimize the appropriate concentration for this primary antibody, McCoy cell monolayers were infected with *C. trachomatis* at a multiplicity of infection (MOI) of 1 for 36 h. Hence, infected cell monolayers were fixed, permeabilized and immunostained with three different 2-fold serial dilutions of the anti-*Chlamydia* LPS antibody (Fig. 1). Following hybridization with the appropriate secondary antibody IRDye 680, plates were scanned and analyzed. Background signals were obtained from non-infected cells, treated as previously described. Results showed that the 1:1000 dilution factor provided higher a.u. values with minimal background and, therefore, was chosen for further experiments (Fig. 1).

Finally, to evaluate any difference in signal intensity between the two IR fluorescent dyes, the performance of these two commercially available secondary antibodies was compared using the same experimental conditions described above for the three model microorganisms. Non-infected cell monolayers stained with primary antibody and either IRDye 680 or IRDye 800 secondary antibodies were used as controls. Plates were scanned and analyzed. Results showed a higher a.u. values for the IRDye 680 antibody compared to IRDye 800 (data not shown). Therefore, the IRDye 680 antibody was chosen and used in all subsequent experiments.

### Quantification of intracellular fast-growing and persistent pathogens

**Fig. 2:**
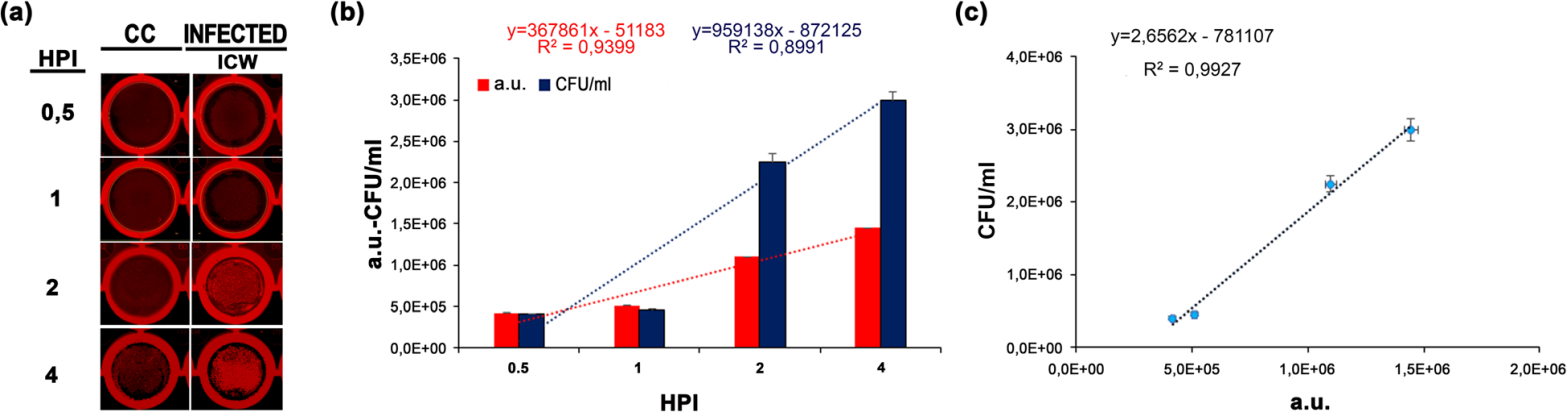
Performance comparison between the ICW assay and standard CFU count upon *S. flexneri* infection. **a** HeLa cell monolayers were infected with the M90 T strain at MOI 100. Non-infected control cells (CC) were used as control. At the selected time points (hours post-infection, HPI), cells were either lyzed for CFU/ml count or immunostained for the ICW assay. Representative images of wells used for the ICW assay from three independent experiments performed in quadruplicate are shown. **b** Histogram shows the mean values ± SD of CFU/ml count (blue bars) and a.u. values (red bars). For both methods, equations and R^2^ values of trendlines are shown. **c** The linear correlation (dotted black line) with relative equation and the coefficient of determination (R^2^ value) are shown

**Fig. 3:**
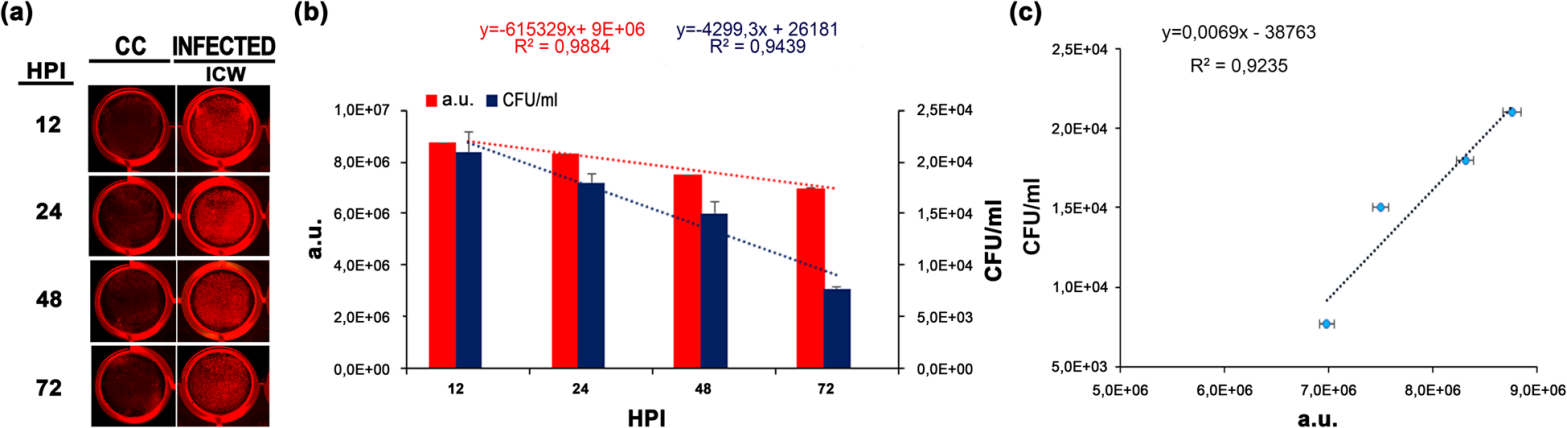
Performance comparison between the ICW assay and standard CFU count upon *E. coli* infection. **a** Hela cells were infected with the LF82 strain at MOI 10. Non-infected control cells (CC) were used as control. At the selected time points (HPI), cells were either lyzed for CFU/ml count or immunostained for the ICW assay. Representative images of wells used for the ICW assay from three independent experiments performed in quadruplicate are shown. **b** Histogram shows the mean values ± SD of CFU/ml count (blue bars) and a.u. values (red bars). For both methods, equations and R^2^ values of trendlines are shown. **c** The linear correlation (dotted black line) with relative equation and the coefficient of determination (R^2^ value) are shown

To determine if the ICW assay could represent a reliable technique for the quantification of intracellular bacteria, gentamicin protection assays were performed using either the M90 T or the LF82 strains. Upon entering host epithelial cells, strain M90 T displays a high and fast replication rate and the ability to spread into adjacent cells [29] while strain LF82 is well adapted to persist within host cells [30]. Quantification of intracellular bacteria was performed using fixed MOIs in time course infection experiments. Indeed, this experimental approach allows us to highlight the different life-style of facultative intracellular bacteria with high and low replication rates. After invasion, *S. flexneri* multiplies and spreads rapidly in cell monolayers, irrespective from the MOI used [29]. Conversely, *E. coli*, as a typical persister, needs at least 24 h to reach an appreciable number of intracellular bacteria [30]. Therefore, the most common MOIs used (MOI 100 for *S. flexneri* and 10 for *E. coli*) were applied for our experiments. After gentamicin addition, cell monolayers were fixed and permeabilized after 1, 2 and 4 h post-infection (HPI) for M90 T and 24, 48 and 72 HPI for LF82 strains (Figs. 2a and 3a). No significant variation in the cell number between non-infected and *E. coli* infected was observed in the three times post infection analyzed, ruling out any bacterial cytotoxic effect. Following staining with specific primary and secondary antibodies, plates were scanned and a.u. values for each well were recorded. Parallel wells of infected cells were lyzed, serially diluted, spot-plated into agar plates and incubated for 16 h at 37 °C for CFU quantification. Results showed that CFU/ml and a.u. values were highly correlated for both strains, as evidenced by a linear relationship, with coefficients of determination of R^2^ = 0.9868 and R^2^ = 0,9174, respectively (Figs. 2b,c and 3b,c). Noteworthy, in the early phase of *S. flexneri* (1 HPI) and late phase of *E. coli* (72 HPI) infections, the ICW technique detected a number of bacteria ranging from 5 × 10^4^ and 1 × 10^3^ bacteria/ml, respectively, indicating a good sensitivity. Moreover, a.u. values showed lower variance compared to the CFU values, highlighting a superior reproducibility of the ICW technique (Figs. 2b and 3b). A certain inaccuracy of CFU count was previously reported [5, 12, 13]; the most important variables that can influence CFU results are related to incomplete lysis of host cells, the detergent used for lysis that can affect bacterial viability, the unreliability of colony counting when colonies tend to coalesce. Moreover, the high variability in the number of CFUs is frequently observed in the enumeration of low amount of bacteria. Therefore, the lack of sample manipulation together with the high specificity of the primary antibody make the ICW approach a technique sensitive, accurate and reproducible for the enumeration of facultative intracellular bacteria.

### Quantification of the obligate intracellular *C. trachomatis* L2 strain 434/Bu

**Fig. 4:**
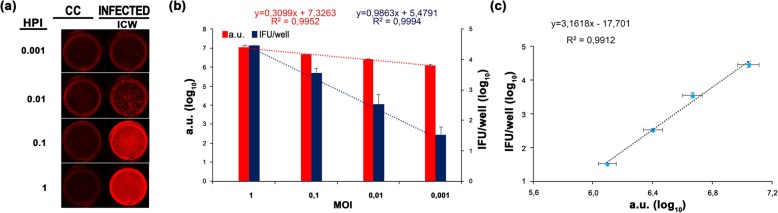
Performance comparison between the ICW assay and standard IFU count upon *C. trachomatis* infection (**a**) McCoy cells were infected with 434/Bu strain at different MOIs, as indicated. Non-infected control cells (CC) were used as control. At 36 HPI, cells were immunostained for IFU microscopic count or for the ICW assay. Representative images of wells used for the ICW assay from three independent experiments performed in quadruplicate are shown. **b** Histogram shows the mean values ± SD of IFU/well count (blue bars) and a.u. values (red bars). For both methods, equations and R^2^ values of trendlines are shown. **c** The linear correlation (dotted black line) with relative equation and the coefficient of determination (R^2^ value) are shown

Next, we evaluated the applicability of the ICW technique for determining the number of IFUs of the obligate intracellular bacterium *C. trachomatis* strain 434/Bu. Due to its absolute requirement of the host, *C. trachomatis* has evolved very efficient means of entering eukaryotic cells [23]. Therefore, to monitor *C. trachomatis* intracellular replication and evaluate ICW sensitivity, cells were infected using an array of different MOIs (Fig. 4). To this aim, McCoy cell monolayers were infected with 434/Bu, fixed and permeabilized at 36 HPI. The first set of wells was stained with the unconjugated anti-*C. trachomatis* LPS antibody (diluted 1:1000) and the IRDye 680 secondary antibody. Plates were scanned and a.u. values for each well were recorded (Fig. 4a,b). Parallel wells of infected cells grown on glass coverslips were stained with the fluorescein isothiocyanate (FITC)-conjugated anti-*C. trachomatis* LPS antibody and mounted onto slides for the IFU enumeration by counting all microscopic fields using a fluorescence microscope at 400× magnification (Fig. 4b). Results showed an excellent correlation between IFU/well and a.u., with a coefficient of determination of R^2^ = 0,9912) (Fig. 4c). Although an old approach, the visual counting by microscope is still the method of choice for quantifying IFUs [18, 22]. However, this method is time-consuming, requires intensive work to be precise, is subjected to investigator bias and restricts large scale analyses. Moreover, several studies report the quantification of IFUs on the basis of a defined number of microscopic fields randomly selected, especially when high MOIs are used [18, 22]. This approach may lead to an under or over-estimation of the total IFUs due to the patchy distribution of *C. trachomatis* on cell monolayer [3]. Therefore, the ICW technique, based on the laser scan of the entire surface of the seeded well on a multiwell plates, eliminates the bias derived by the number of analyzed fields, the variability among investigators and allows the application for high-throughput analyses. To overcome the drawbacks of the microscope count, several methods have been developed for quantifying *Chlamydiaceae* mainly based on fluorescence, colorimetric and real-time-PCR approaches [16–18, 31]. Among those, quantification of immunostained *C. trachomatis* cells by flow cytometry is highly suitable, being extremely sensitive for the huge number of cells that can be analyzed and has negligible variance among investigators [18, 32]. Flow cytometry was also applied for green fluorescent protein (GFP)-tagged bacteria. Although appropriate, this approach is limited to the number of tagged strains, the variable level of GFP expression and the resistance of selected strains to the GFP-associated oxidative stress [17, 33]. However, not all labs are equipped with a flow cytometer whose acquisition and maintenance costs are considerable. For this reason, methods involving fluorescence plate reader scanners that record the number of inclusions in flat-bottom multiwell plates are currently a valuable alternative for quantifying *Chlamydiaceae* [16, 34, 35].

## Conclusions

In summary, the automatic, fast, reliable and high-throughput laser scan-based technique ICW assay was applied for the first time for quantifying intracellular bacteria in in vitro infection models. We evaluated this assay by testing three different microorganism models, two facultative intracellular pathogens, the fast-growing *S. flexneri* and a persistent strain of *E. coli*, as well as the obligate intracellular pathogen, *C. trachomatis*. Signal intensity values achieved from the ICW assay were positively correlated with standard quantification methods (i.e. CFU and IFU counts), allowing to infer the CFU/ml from the regression equations with a difference of about 25% with respect to the actual CFU/ml numbers. Based on the microorganism to be studied, the appropriate MOI, time of incubation and antibody should be chosen in order to achieve CFU and a.u. values as close as possible to a linear relationship. This method was found exquisitely suitable in measuring the changes in the number of intracellular bacteria over time post-infection and MOIs, being able to determine differences ranging from one to four logs. Unlike the classical counts, the ICW assay displayed values with negligible variance in each experiment, indicating high accuracy and reproducibility. Moreover, the protocol is simple and requires 4 h-work; it can be paused and processed the day after or being re-analyzed more than 1 month later. Moreover, the software is easy to use and intuitive. This high-throughput assay, allowing the analysis of up to 576 well in parallel, is suitable for the study of a broad range of intracellular pathogens characterized by different life-styles, since no tagging is required for bacteria. However, it is important to note that the availability and specificity of the primary antibody are the major limitation of the ICW assay. In addition, some pathogens use phase variation to modify surface antigenicity as a virulence strategy during and after invasion of eukaryotic cells [36]. Indeed, the antigenic modifications dramatically change the signals of the ICW. Therefore, the choice of the primary antibody should take this aspect into account and, in those cases, it would be advisable to use a second primary antibody in parallel to verify possible antigenic modifications. Further experiments are required to broaden the application of this assay in the research field (i.e. the use of molecular probes to study the intracellular trafficking of pathogens within host cells) and to open its use for diagnostic purposes.

## Methods

### Materials and reagents

Culture media, Dulbecco’s modified Eagle’s medium (DMEM), fetal bovine serum (FBS), fetal calf serum (FCS) and trypsin−EDTA were purchased from Gibco Corp (Euroclone, Italy) whereas HEPES buffer and cycloheximide solution from Sigma-Aldrich, St. Louis, USA. Primary antibodies: rabbit anti-*S. flexneri* serotype 5 (cat. no. 295040, Denka Seiken, Japan), goat anti-*E. coli* (cat. no. ab13627, Abcam, UK), mouse monoclonal antibody anti-*Chlamydia* LPS (Clone B410F) either unconjugated or FITC-conjugated (cat. no. MA1–7338 and MA1–7339, respectively, Thermo Fisher, Italy). Commercially available secondary antibodies: goat anti-rabbit IRDye 680RD/IRDye 800CW, donkey anti-goat IRDye 680RD/IRDye 800CW and goat anti-mouse IRDye 680RD/IRDye 800CW (LI-COR Biosciences, Nebraska, USA; https://www.licor.com).

### Bacterial strains and cell lines

The *S. flexneri* 5a strain M90 T (ATCC BAA-2402), *E. coli* strain LF82 and the obligate intracellular pathogen C. *trachomatis* serovar L2 strain 434/Bu (ATCC VR-902B) were used in this study. *S. flexneri* M90 T and *E. coli* LF82 strains were propagated onto Congo red and MacConkey agar plates, respectively. For infection experiments, both strains were grown to exponential phase in Luria Bertani (LB) broth to an optical density at 600 nm (OD_600_) corresponding to 1 × 10^8^ and 5 × 10^8^ colony forming units (CFU)/ml, respectively. *C. trachomatis* strain 434/Bu was propagated in mouse fibroblasts McCoy cells (ATCC CRL-1696) as previously described [3]. The infectious titer, corresponding to 3 × 10^8^ IFU/ml, was assessed by direct immunofluorescence assay. Briefly, sub-confluent McCoy cell monolayers grown on glass coverslips in 24-well plates were infected with 10-fold serial dilutions of bacterial stock, incubated for 36 h at 37 °C, fixed with methanol and stained with FITC-conjugated monoclonal antibody anti-*C. trachomatis* LPS. The total number of *C. trachomatis* IFU was enumerated by counting all microscopic fields using a fluorescence microscope (400× magnification) [3].

The human cervical HeLa cells (ATCC CCL-2) and McCoy cells were used for infection experiments. Both cell lines were routinely grown in DMEM supplemented with 10% (v/v) heat-inactivated FBS and FCS, respectively, at 37 °C in humidified atmosphere with 5% CO_2_.

### Infection assays

For infection experiments, HeLa and McCoy cells were seeded at 1 × 10^5^ and 2 × 10^5^ cells per well, respectively, directly or on standard glass coverslips in 24-well plates and grown for 24 h at 37 °C in humidified atmosphere with 5% CO_2_. Hela cell monolayers were infected with strains M90 T and LF82 at MOIs of 100 and 10, respectively, and centrifuged at 2000×*g*, 10 min, 37 °C. After incubation, 45 min for M90 T and 2 h for strain LF82, monolayers were extensively washed with phosphate-buffered saline (PBS) and further incubated with cell culture medium supplemented with 100 μg/ml gentamicin to kill extracellular bacteria [30, 37]. Three different time points were selected for intracellular bacteria quantification: 1, 2, and 4 HPI for strain M90 T and 24, 48, and 72 HPI for strain LF82. Sub-confluent McCoy cell monolayers were infected with strain 434/Bu for 36 HPI at MOI 1, 0.1, 0.01 and 0.001.

### CFUs and IFUs counts

After the indicated incubation period, cell monolayers infected with facultative intracellular bacteria were washed twice and lyzed with 0.1% Triton X-100 in PBS. Lysates containing recovered intracellular bacteria were diluted, spot-plated onto LB-agar plates in triplicate and incubated overnight at 37 °C. The day after, the number of CFU/ml was recorded. At 36 HPI, infected McCoy cells were fixed with methanol and stained with FITC-conjugated anti-*Chlamydia* LPS antibody, following manufacturer’s instructions. The number of *C. trachomatis* IFU/well was determined by counting all microscopic fields using a fluorescence Leica DM5000B microscope (Leica) at 400× magnification [3].

### ICW staining and processing

Infected cells were washed six times with PBS to remove cell-bound killed bacteria, fixed with 4% paraformaldehyde in PBS for 10 min at room temperature (RT) and permeabilized with 0.1% Triton X-100 in PBS for 8 min at RT. Following incubation with Odyssey Blocking Buffer for 30 min at RT, cell monolayers were incubated with the specific primary antibodies, diluted in Odyssey Blocking Buffer, for 1 h at RT and washed three times with PBS containing 0.1% Tween-20. Afterward, appropriate IR conjugated secondary antibodies were added and incubated for 1 h at RT. Finally, cell monolayers were washed three times with PBS containing 0.1% Tween-20 and wells were allowed to dry. Non-infected cell monolayers stained either with both antibodies or only with secondary antibodies were used for subtracting unspecific and/or background fluorescent signals. Multiwell plates were analyzed on a laser scanner Odyssey infrared imaging system (LI-COR Biosciences). The system provides specific wavelength of excitation (either the IR 700 or 800 channels) and, therefore, records the amount of specific fluorescence signals emitted by the samples in the multiwall by an Avalanche Photodiode Detector (APD); APD converts the analog signal to a digital scale between 0 and 4,194,303 fluorescence a.u. The Odyssey system was used in default parameters, resolution set to 169 μm in auto scan mode; images and a.u. values from each well were acquired using the LI-COR Image Studio software. Recorded a.u. values were then exported into an Excel spread-sheet (Microsoft, version 1808) and analyzed.

### Data analysis

Three independent experiments were performed in quadruplicate and data are reported as mean ± standard deviation (SD). Normal distribution was determined by the Shapiro–Wilk test (STATA 12, Texas, USA). The correlations between a.u. values and CFU/ml or IFU/well were analyzed by calculating the coefficient of determination (R^2^) by Excel spread-sheet (Microsoft, version 1808). All data analyses were performed using GraphPad Prism (GraphPad Software, Inc., San Diego, CA).

## Data Availability

All data generated or analyzed during this study are included in this published article.

## Abbreviations

a.u.: Arbitrary units
AIEC: Adherent-invasive *E. coli*
APD: Avalanche Photodiode Detector
*C. trachomatis*: *Chlamydia trachomatis*
CFU: Colony forming units
DMEM: Dulbecco’s modified Eagle’s medium
*E. coli*: *Escherichia coli*
FBS: Fetal bovine serum
FCS: Fetal calf serum
FITC: Fluorescein isothiocyanate
GFP: Green fluorescent protein
HPI: Hour post-infection
ICW: In-Cell Western
IFU: Inclusion forming units
IR: Infrared
LB: Luria Bertani
LPS: Lipopolysaccharide
MOI: Multiplicity of infection
OD: Optical density
PBS: Phosphate-buffered saline
RT: Room temperature
*S. flexneri*: *Shigella flexneri*
SD: Standard deviation
